# Pennsylvania Hospital

**Published:** 1838-05-09

**Authors:** 


### CLINICAL LECTURES.

### PENNSYLVANIA HOSPITAL.

*Saturday, April* 7th. At 11 o'clock DR. HARRIS commenced: I propose this morning to make a few remarks on affections of the bones; they are subject to the same diseases as the soft parts, and are liable to inflammation, accompanied by similar symptoms, as heat, redness, pain, &c., which may terminate in suppuration, ulceration or caries, and gangrene or necrosis. We have also affections of a malignant character, such as osteo sarcoma.

Caries occurs in the epiphyses, bodies of the vertebræ, and in the flat bones; whilst on the other hand, necrosis is found in the shafts of the long bones. When inflammation takes place in the latter, it is very apt to terminate in its death. The periosteum does not die with it, but is detatched, and commences the secretion of a new bone, by which the old bone is encased. Frequently the dead bone occasions much constitutional irritation, which requires its removal. This must not be done until its separation is entire, as it would excite too much irritation and pain. When, however, this separation has occurred, the sequestrum is to be taken away by cutting through the soft parts, applying a trephine to the new bone, when the opening is not sufficiently large, and removing it with the forceps.

In caries no restoration of the bone takes place, as in necrosis, by the secretion of a new bone by means of the periosteum. If a flat bone be the subject of disease—as for instance the frontal, or parietal—a ligamentous substitute is formed.

When caries occurs in the joints, producing white swelling, it commences in the synovial membrane, which becomes thickened; a gelatinous fluid is secreted; the disease extends to the cartilages and the heads of the bones concerned in the articulation, rarely commencing in the cancellated structure.

In the beginning this disease, arising as it mostly does from a sprain, from cold, or from mechanical violence, is curable. If called early, you must insist upon an absolute state of rest. Dr. Physic, who made so many important discoveries in regard to the treatment of affections of the bones, instantly ordered the patient to his bed, to remain there for two or eight weeks, or even a longer period. Our first remedies are to be of the depleting kind, and are most important. If called later, and the limb is greatly swollen, the patient suffering from the pain arising from the amount of fluid, or, perhaps, pus secreted in the joints, a question has arisen as to the propriety of opening it. The constitutional irritation arising from such distention is very great. I would prefer promoting the absorption of the fluid in the joint, which not unfrequently succeeds. If we fail in such efforts, it is more judicious to tap the joint, than allow the patient to be worn out by the pain and irritation, caused chiefly by the distention.

In addition to the causes already enumerated, a peculiarity of constitution and condition of the system may produce the disease. Many surgeons have considered syphilis as another cause. This I doubt, for when it has been alleged to have arisen from this affection, it has invariably been found to have taken place after the use of mercury.

Although, perhaps, not exactly in place, I would remark, that owing to the fatality of the venereal disease among the British soldiers in the Peninsula, Rose, Ferguson, Hennen, and Guthrie, were led to pursue the Portuguese plan of treating it without mercury, and in not a single instance was it followed by diseased bones. When Sir James Mac Grigor, the director general, received the reports from the British army surgeons connected with the army of Spain, he issued a circular directing all the surgeons to make comparative trials of the two plans of treatment, and all concurred in reporting the same results.

Fracartorius, an eminent physician of Naples, who wrote about the termination of the fifteenth century, remarks: "During my practice, the venereal disease has in some degree changed its character. When I first treated this affection, it never invaded the bones, but now these symptoms are by no means uncommon. This new feature in the disease, I attribute to the new remedy, which is mercury." It was about this period that mercury was introduced as a medicine.

[Dr. Harris now introduced a patient affected with caries of the scapula, and continued:]

The man presented before you fell, in June last, from a two story house, and severely contused the acromion and spine of the scapula. It was followed by pain and heat of the injured part, inability to move the shoulder joint, or to use the arm, without aggravating the pain of the shoulder. He was brought into the hospital in this condition in August, about two months after the accident. In January he was seized with erysipelas of the part covering the scapula and shoulder, accompanied by great constitutional disturbance. The disease yielded to the ordinary remedies, but left the patient in a state of extreme prostration. In three weeks afterwards, and before he had entirely recovered his strength, he was attacked with the same disease, and which was repeated every few weeks, until his life was placed in great jeopardy. In this instance free incisions were made to the bone, which afforded prompt relief of all the local and constitutional symptoms. These incisions disclosed the state of the bone, and determined us to remove the caries.

Portions of the scapula have been often removed, and Cheselden reports a case, where he removed the entire bone.

Excision of joints, too, is now frequently performed to relieve patients from the destructive effects of caries. Terrible as this operation may appear, it is attended with a degree of success which has proved highly encouraging. The great recommendation of excision, is, that it saves to the patient a useful limb.

The objections to the operation are, the difficulty in performing it, the danger, and the useless condition of the limb afterwards.

The difficulty of an operation should never constitute an objection, if it can be done with comparative safety. The success of White, of Manchester, Dupuytren, Syme, of Edinburgh, and others have ranked it among the standard operations.

In sixteen operations of carious elbow joint, on which Syme performed the operation of excision, all recovered except three. This is much greater success than attends amputations. In Paris one half die after amputations, and in this city rather less than one fourth.

The frequent cause of death after amputation, arises from the formation of metastatic abscess. This disease seems to be caused by a disturbance of the functions of the system, by the loss of so considerable a portion of the body. The objection to opening the joint should not be deemed valid, as it is already in this condition by the progress of the disease.

So far is the limb from being rendered useless by this operation, that it, in a majority of cases, performs its various offices almost as perfectly as before the injury was inflicted. The only instance in which I have excised a carious joint, was in this hospital three years ago. In this case, the whole of the elbow joint was in a state of caries. The operation was successfully performed, and the patient now enjoys perfect health, and the hand and arm can perform all the ordinary offices of a hard working woman.

In the case before you, the shoulder joint is stiffened, and the structure around it thickened. After careful examination, however, I am inclined to think that it is not affected by caries. After the disease of the scapula is exposed by dissection, then the condition of the joint may be accurately ascertained. Should the caries extend to the glenoid cavity of the scapula, and to the head of the humerus, I am prepared to excise the whole articulation.

[Dr. Harris now placed the patient in a horizontal position on his sound side, and made an incision, commencing near the base of the scapula, and along its spine to the acromion process. The muscles were detached from either side of the spine of the scapula, which fully exposed the disease, which occupied two thirds of its extent. The caries was removed with the cutting plyers and chisel. A portion of the acromion not being involved in the disease, was preserved. The caries, happily, did not extend to the joint.]

---

LITHOTRIPSY.

*Wednesday*, April 18th. At 11 o'clock, Dr. Randolph, commenced: I propose, gentlemen, today, to call your attention to a case of stone in the bladder, and I shall exhibit to you presently a little patient, aged thirteen years, upon whom I shall attempt to perform an operation, which, at this time, claims the serious attention of the surgical profession. This case is rendered more interesting from the fact that, eleven years ago, I removed a stone from this boy's bladder, when he was but two years of age, by means of the operation of lithotomy. Here is the calculus which I removed whole and entire. It is composed principally of uric acid. The incisions healed, after the first day, by the first intention; after that time no urine passed through the wound. This is the third or fourth case, during my practice, in which I have known the recurrence of a stone in the bladder after its complete extraction, by means of the operation of lithotomy.

This boy was brought, about four weeks since, into the house, in a state of great suffering. His bladder was in a very irritable condition, and he endured a great deal of pain, particularly after voiding his urine. He also experienced a frequent desire to pass his urine, and an inability to hold it for any length of time. When he commenced urining, the water, sometimes, stopped suddenly, and he was induced to strain violently, but ineffectually; after a short time, however, it would recommence flowing. Some blood and mucus were occasionally discharged through the urethra. His efforts at straining were so violent as to produce *prolapsus ani;* this is not an uncommon attendant on calculus in the bladder.

For the relief of this painful malady surgeons were, until very recently, in the habit of cutting open some part of the bladder, and extracting the stone by means of the forceps. I shall not detain you now with a description of the various cutting operations which have been employed for the removal of stone in the bladder. The one most generally preferred of late years, and which is most frequently performed in this city, is called the *lateral* operation of lithotomy. It is done in this manner. A grooved staff is introduced into the bladder, through the urethra, and an incision is made in the perineum, down to the membranous part of the urethra, which is divided, laying bare the groove of the staff; next the prostate gland, and neck of the bladder, are to be divided laterally, either by a knife or the gorget, and the stone extracted by a pair of forceps. Notwithstanding, in this country, the operation of lithotomy has been attended with the most extraordinary success, it must be conceded that it is one of the most horrible, terrifying, and hazardous operations which the surgeon is called upon to perform. It is replete with danger, and liable to numerous objections. The patient in some instances dies of hemorrhagy from vessels divided out of sight and out of reach. Convulsions occasionally follow, under which he may succumb, or he may die from great prostration, or the shock his nervous system receives at the time of the operation. When the patient escapes these immediate dangers, he is liable to the occurrence of peritoneal inflammation some few days subsequently, the most common cause of death after this operation. The calculus, too, when soft, during its extraction, may break into pieces, rendering the operation tedious, and increasing its dangers. In such cases, too, the disorder is likely to return, from some portion of the stone remaining in the bladder, and acting as a nidus for after concretions.

Another inconvenience attending the operation of lithotomy, is the liability to incontinence of urine. This is particularly the case in females, especially adults, and is an evil of such magnitude, and so distressing, that you had almost as well suffer your patient to die as to expose her to it, for under such circumstances life is not desirable. In the male, too, the incisions in the perineum sometimes do not heal, and you have established a *fistula in perineo*, a most difficult condition to relieve. Now when the patient is lucky enough to escape all these inconveniences, and the case progresses successfully and happily to a cure, he often is condemned to lie for several weeks in his bed with all the clothing about him saturated with water.

To avoid all these inconveniences and hazards, the ingenuity of several European surgeons became directed to the invention of instruments, by means of which, a stone in the bladder might be gotten rid of without any cutting operation. It is now about sixteen years since the news reached this country, that Mr. Civiale, of Paris, had invented instruments, by means of which he was enabled to remove a calculus, without any incision. In 1824, Mr. Civiale submitted to the French Royal Academy of Medicine, an account of his plan of operating, together with his instruments. A committee was appointed, by this institution, to examine into its merits. It consisted of the celebrated Surgeons Baron Percey, and Mr. Chaussier. These gentlemen examined carefully the claims of this operation to favour, and in their report pronounced it to be "glorious for French surgery, honorable to its inventor, and consoling to humanity."

It is proper that I should mention to you that about this period, or perhaps previous to it, Mr. Leroy d'Etiolle, of Paris, appears, by a singular coincidence, to have been engaged in the same manner Mr. Civiale had been, and he also produced instruments very similar to Civiale's, and which operated upon precisely the same principles. The idea of destroying a stone in the bladder did not, however, originate with either of these gentlemen; it had been conceived a long time previous, and instruments had been devised for the purpose, drawings of which we now have.

Mr. Gruithuisen, of Bavaria, preceded both Mr. Leroy and Mr. Civiale in proposing a method of destroying a calculus in the bladder.

Be this though as it may, from this period Civiale went on performing his operation with immense success, and there is indisputable evidence of his having effected a great number of cures. His reputation consequently soon became very great, and persons came from a great distance to obtain relief at his hands. As might be expected, his method now met with considerable opposition from many of the other surgeons who were unwilling to admit his brilliant success, and would not be convinced that his operation presented any extraordinary claims to their consideration, or that it would be destined to prove preeminently beneficial to suffering humanity in removing stone from the bladder. These gentlemen called in question the accuracy of Mr. Civiale's statements; they alleged, and, I am sorry to say, not without some reason, that he had exaggerated the number of his cases, as well as the success of his operations. Admitting this to be the fact, still no one can doubt that he has performed a sufficient number of cures to secure him lasting fame and celebrity, and to establish, incontestibly, the great value of the operation.

As soon as Civiale's operation became generally known in Europe, and its successful results were clearly established, other surgeons directed their attention to the same subject, and they succeeded most happily in inventing instruments less complicated than his, and by means of which a calculus may be destroyed in the bladder more speedily, more safely, and quite as effectually. I allude particularly to the instrument invented by Mr. Jacobson, of Copenhagen, which he calls the "Brise-pierre-articulé," and the instrument invented by Baron Heurteloup, called by him the "Percuteur Courbe." It is undeniable that these instruments possess considerable advantages over Civiale's, which they have almost entirely superceded. Mr. Civiale indeed admits their superiority, and notwithstanding he occasionally uses his own instruments, he has modified the "Percerteur Courbe" of Baron Heurteloup, and does not hesitate to employ it upon suitable opportunities.

[Here Dr. Randolph proceeded to exhibit, and describe to the class, the various instruments employed in Lithotripsy. He first showed and explained the *lithontripteur* of Civiale. He stated that he had employed this instrument in his first four cases, although he completed each of these operations with the *brise-pierre-articulé* of Mr. Jacobson. He is of opinion that in some cases the *lithontripteur* may prove of great service, as where an unusually hard calculus exists. One or two perforations would diminish the strength of the stone so much as to cause it to be readily crushed by either Baron Heurteloup's, or Mr. Jacobson's instrument.

He next exhibited the *brise-pierre-articulé* of Mr. Jacobson, of Copenhagen. This instrument he has used in eighteen cases, and prefers it to all others. It is perfectly simple, possesses great strength, and is as easily introduced into the bladder as an ordinary catheter. Objections, he stated, had been made against this instrument, but he conceived without any valid foundation. It has been stated that it was liable to pinch the bladder; this is far from being the fact, he said. Dr. R. replied at length to this, and also to several other objections which have been urged against the instrument.

Dr. R. next showed Heurteloup's *percuteur courbe à marteau*, and mentioned the great success which had attended the use of this instrument. He stated that he had used it in one case only; the last one he had operated on, and which was reported in the Examiner, No. 7, p. 116.

Some instruments for extracting fragments of stone from the urethra were also exhibited, and commented upon.]

Dr. Randolph resumed:

Soon after it became known in this country that Civiale had succeeded in removing calculi from the bladder without a cutting operation, the surgeons here very readily and willingly adopted the idea; they provided themselves with the best instruments that they could procure, and set about performing the operation. In consequence, however, of their instruments being made by persons wholly unaccustomed to their construction, and from the imperfect descriptions our surgeons were enabled to give, and in consequence also of the surgeons not having sufficiently studied the operation, and not having practised it upon the dead subject previously to applying it to the living one, I am sorry to say that these first efforts were entirely unsuccessful, and that they did not, in this city, succeed in a single instance. In consequence of this failure they became discouraged, and thought that the merits of the operation had been much exaggerated, and that it could only succeed in very rare instances. I, in common with my professional brethren, adopted the most favourable impressions respecting the operation, and having witnessed several of the unsuccessful efforts, I determined to qualify myself in every possible way for the performance of the operation. My situation at that time, as surgeon in the Alms-House Infirmary, afforded me every facility for practising the operation upon the dead subject, and I embraced every opportunity of putting a stone into the bladder and destroying it by means of the instruments. Soon after this, I procured from Paris, a complete set of Civiale's instruments, and also the "Brise-pierre-Articulé" of Mr. Jacobson, and proceeded to the performance of my first operation in September, 1832. My patient was named Augustin, a French cook, well known in this city, aged 50 years. He had been afflicted with the stone for several years; it was large and composed principally of the phosphate of lime. I performed seven operations upon this patient, four by means of the Lithontripteur of Mr. Civiale, and three by means of the Brise-pierre of Mr. Jacobson. His urethra was very large, and he passed through it fragments of stone of an almost incredible size, with but little inconvenience. These operations were performed in the presence of Drs. Physick, Horner, La Roche, Rush, and other medical gentlemen. This patient remains perfectly well at the present period; of this, he assured me a few days since.

I do not intend, gentlemen, to detain you by detailing at length all my cases of Lithotripsy. Seventeen of these I have reported in the American Journal of the Medical Sciences. Since the last report I have performed the operation successfully in two other instances, making *nineteen* cases. Three of these patients were inmates of this house.

My sixteenth case was Dr. Silas Tompkins, of New Bedford, Massachusetts, who came to this city to put himself under my charge, and to be in this institution. This gentleman suffered also from paraplegia. He published an account of his case in the Boston Medical and Surgical Journal, on his return home, and declared himself to be perfectly cured.

It has been urged, as an objection to the operation of Lithotripsy, that it is only partially applicable, and that it can be performed, advantageously, in a limited number of cases only. To this I may reply, that, out of upwards of twenty cases of stone, occurring in adults, which, within the last six years have occurred in my practice, I have found two only to which Lithotripsy was not applicable. I therefore am of the opinion that in adults, the operation may be adapted to eighteen cases out of twenty of stone. Another objection which has been advanced against Lithotripsy is, that fragments of the calculus are liable to remain in the bladder and form the nucleus of future stones. But I contend that the presence of the smallest fragment may be detected not only by sounding, but also by the feelings of the patient. The feelings of the patient may indeed be received as a pretty certain test of a perfect cure. When they say to you, "I am quite well," such is generally the case. I have rarely found it otherwise. Of course you should not rely upon their assertion, but satisfy yourself always by careful and repeated sounding. Indeed in many instances the smaller the fragment is, the more acute the suffering appears to be. The stone in such cases lodges at the neck of the bladder, and intense pain accompanies every effort to pass the water. When large the calculus falls to the fundus of the bladder, and does not obstruct so much the flow of urine.

It has been said too by the opponents of Lithotripsy, that you are unable to break up a very hard calculus, as for example an oxalate of lime. In refutation of this I will state to you that in May, 1836, Mr. William Reynolds, of Bedford, Pennsylvania, came to this city to consult me for stone in the bladder. He had been laboring under the affection for four years, and had suffered a great deal of pain. His bladder was very irritable and contracted, and he was unable to retain more than a gill of urine, and during the night he passed it involuntarily. The only relief he found for his great agony, was to lay on his back, elevating his legs, so as to throw the stone on the fundus of the bladder. On no occasion had he passed any sabulous matter. Now, in the soft calculi more or less sand is most generally mixed with the water voided—with the hard none. I introduced a sound, and found a very large calculus, and from the ringing sound the percussion gave, as well as from the rugged eminences with which I felt it studded, I was convinced that it was a mulberry calculus—the hardest description of calculus. I told him that I was not willing to cut him for stone, as his bladder was, I feared, extensively diseased. I also told him that I was not sure I could crush it, as the possibility of doing so had been denied by respectable authority, but that I would try. On the 22d of May, in the presence of several medical gentlemen, I commenced the operation. I introduced the instrument of Jacobson into his bladder, caught the stone immediately, and wrapping a towel round the handle, I proceeded gradually turning the screw. In a few seconds I had the satisfaction of finding the stone give way. The operation was repeated afterwards several times, until every particle of the stone was removed. The stone was subsequently analysed, and found to be an oxalate of lime.

I have already mentioned to you that at the time I commenced my operations, for Lithotripsy, in consequence of the repeated failures which had attended the previous efforts to perform this operation, many surgeons became discouraged, and were disposed to think that the value of the operation had been much overated. I am happy to say, however, that it has been taken up by other gentlemen in this city, and elsewhere, and they have performed it with great success. Dr. N. R. Smith, of Baltimore, has lately reported several interesting cases of stone, some of them occurring in very young children, upon whom he performed the operation of Lithotripsy with the most happy results. The operation of Lithotomy, however, in children is comparatively so safe, that a substitute is hardly desirable.

I shall now introduce the little boy upon whom I propose to operate. His bladder and urethra are very irritable. Some days since I introduced the Brise-pierre into his bladder in order to see how he would bear it; I caught the stone and broke it once. He has passed out these fragments, which I here show you. Even this solitary operation gave him some relief.

[Dr. Randolph now proceeded to operate. He introduced the instrument of Mr. Jacobson into the bladder and caught the stone three times, and crushed it with great ease. The boy was afterwards placed in a warm hip bath and fifteen drops of laudanum administered to him. Upon being questioned, closely, he declared that it did not cause him much pain. When the operation is completed we shall report it in full.]

